# A Critical Analysis of the Impact of Etoposide as a Topoisomerase II Inhibitor in Cervical Cancer Treatment: A Review

**DOI:** 10.1002/cam4.71482

**Published:** 2026-03-02

**Authors:** Nikitha Kotian, Yashaswini Reddy, Padmini Pai, Babitha Kampa Sundara

**Affiliations:** ^1^ Manipal School of Life Sciences, Manipal Academy of Higher Education Manipal Karnataka India; ^2^ Department of Biophysics Manipal School of Life Sciences, Manipal Academy of Higher Education Manipal Karnataka India

**Keywords:** cervical cancer, combination therapy, etoposide, sensitivity, topoisomerase II

## Abstract

**Background:**

Etoposide is a semisynthetic derivative of podophyllotoxin and an FDA‐approved topoisomerase II inhibitor that induces DNA strand breaks leading to cancer cell death. Cervical cancer remains the fourth most common cancer among women worldwide, with platinum‐based chemotherapy constituting the standard of care. Although etoposide has demonstrated broad anticancer activity, its role in cervical cancer therapy is considered secondary and is mainly explored in combination regimens.

**Methods:**

This review summarizes published clinical and preclinical studies evaluating the use of etoposide in cervical cancer, both as monotherapy and in combination with other chemotherapeutic agents. Emphasis is placed on treatment regimens, disease stages, routes of administration, limitations, and emerging strategies aimed at improving therapeutic outcomes.

**Results:**

The etoposide has been administered orally or intravenously and has shown clinical benefit primarily when used in combination therapies, including cisplatin, topotecan, mitomycin, epirubicin, doxorubicin, vincristine, cyclophosphamide, bevacizumab, and adriamycin. Its application is mainly observed in FIGO stage IA2–IB2, as well as in recurrent or metastatic cervical cancer. However, platinum‐based regimens, particularly cisplatin or carboplatin combined with paclitaxel, topotecan, fluorouracil, or bevacizumab, remain superior in efficacy. Limitations such as drug resistance and systemic toxicity have restricted the widespread use of etoposide. Recent research has focused on nanocarriers, dual inhibitors, and polymeric implants to enhance its therapeutic index and reduce adverse effects.

**Conclusion:**

While etoposide is an effective topoisomerase II inhibitor with proven anticancer activity, its role in cervical cancer remains adjunctive rather than primary. Combination‐based strategies and advanced drug‐delivery systems hold promise for improving its clinical utility. Continued research is essential to overcome resistance and toxicity, potentially expanding the therapeutic relevance of etoposide in cervical cancer management.

## Graphical abstract

Etoposide mediated topoisomerase II inhibition in Cervical Cancer.

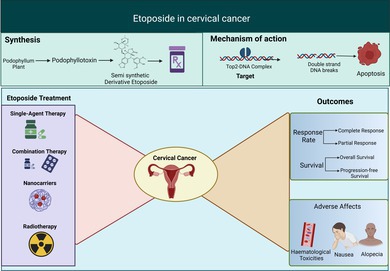

**Abbreviations**
ADC: antibody‐drug conjugate
CCRT: concurrent chemotherapy and radiation therapy
Cdk2 kinase: cyclin‐dependent protein kinase 2
CI: combination index
CR: complete response
CSC: cancer stem cells
CSS: cancer‐specific survival
DRI: dose reduction index
DSBs: double‐strand breaks
EBRT: external beam radiation therapy
EP: etoposide and cisplatin
FFS: failure‐free survival
FIGO: The International Federation of Gynecology and Obstetrics
GnRHa: gonadotropin‐releasing hormone agonist
HDAC: histone deacetylases
HPV: human papilloma virus
IC50: inhibitory concentration 50
IEP: ifosfamide, etoposide, and cisplatin
IMRT: intensity‐modulated radiotherapy
LACC: laparoscopic approach to cervical cancer
LVSI: lymphovascular space invasion
MEP: mitomycin C, etoposide, and cisplatin
MEPA: mitomycin C, etoposide, cisplatin, and epirubicin
NAC: neoadjuvant intraarterial chemotherapy
NACT: neoadjuvant chemotherapy
NECC: neuroendocrine cervical cancer
OS: overall survival
PD: progressive disease
PEITC: phenethyl isothiocyanate
PFS: progression‐free survival
P‐gp: P‐glycoprotein
POEOMA: Poly (ethylene glycol) monomethacrylate
PPC: peak plasma concentrations
PR: partial response
RFS: recurrence‐free‐survival
RNF4: RING finger protein 4
SCCC: small cell cervical cancer
SCNEC: small cell neuroendocrine cervical cancer
SD: stable disease
SFN: sulforaphane
SHAPE: simple hysterectomy and pelvic node assessment
TFF3: trefoil factor 3
Topo: topoisomerase
VAC/PE: vincristine, doxorubicin, cyclophosphamide, cisplatin, and etoposide

## 1 Introduction

Etoposide (4′‐demethyl‐epipodophyllotoxin‐9 (4,6‐O‐ethylidene‐β‐D‐glucopyranoside)) is a semisynthetic compound derived from podophyllotoxin [1]. Podophyllotoxin is a substance extracted from plants belonging to the genus *Podophyllum*. Aqueous extracts from the plants *Podophyllum peltatum* and *Podophyllum embodi* were used for therapeutic purposes hundreds of years ago by American Indians and Natives in the Himalayan Mountain region. When applied topically, Kaplan reported that oil containing podophyllin selectively destroyed condyloma acuminatum (genital warts). Podophyllin was removed from the United States Pharmacopeia in 1942 because of its severe toxicity. Various podophyllin derivatives have been synthesized by Sandoz Laboratories since 1963, and the two most successful derivatives are etoposide and teniposide [2].

**FIGURE 1:**
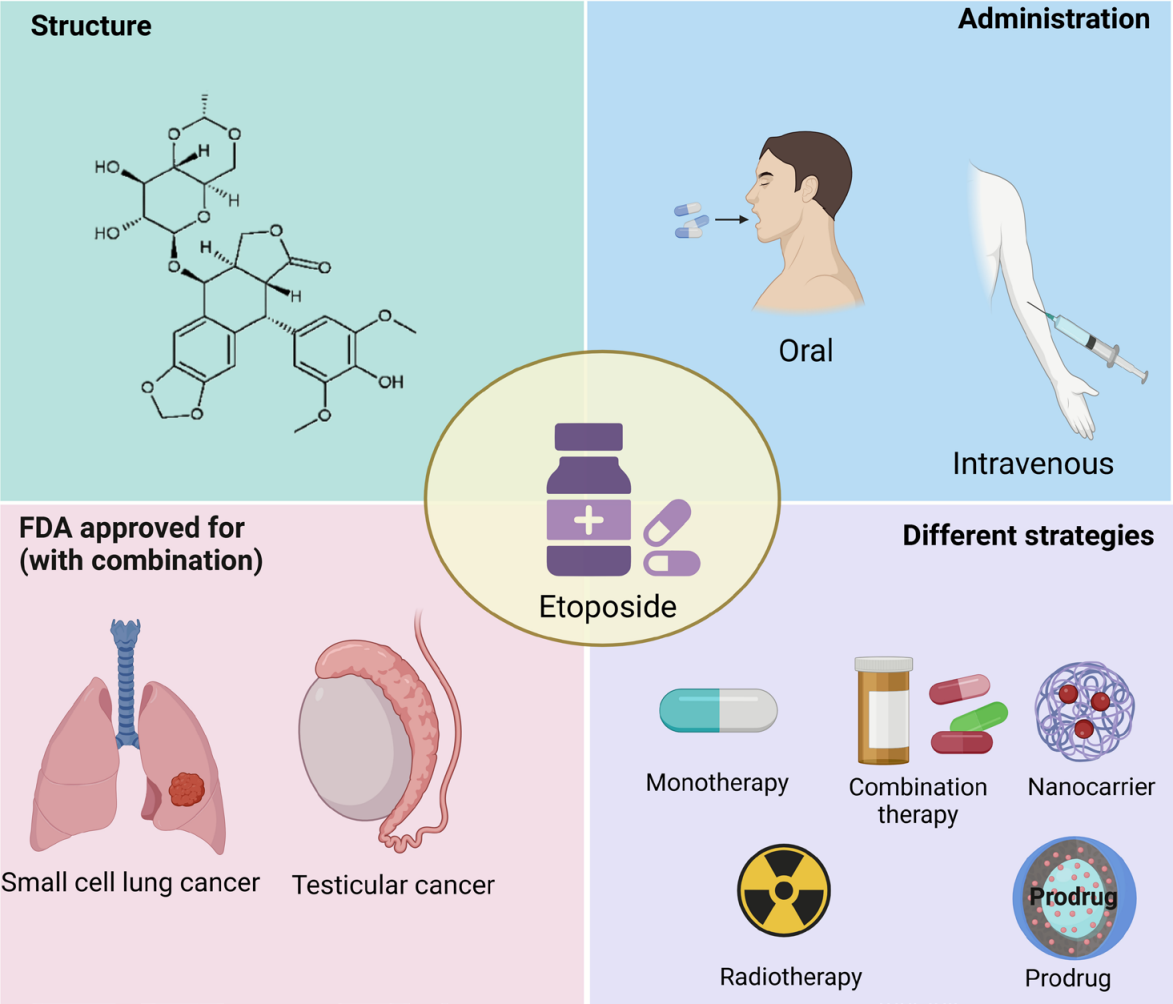
Etoposide: Structure, administration, FDA approval for various cancers, and commercial names.

Etoposide is an antineoplastic agent and has been used both alone and in combination to treat many cancers, such as testicular tumors, Kaposi's sarcoma, and small cell lung carcinoma [3]. Etoposide has been used in clinical studies since 1971. It has been demonstrated to have antineoplastic effects on lung cancer, acute myeloid leukemia, and non‐Hodgkin's lymphoma. Etoposide was approved by the FDA in 1983 for treating small cell lung cancer and testicular cancer with other drugs [4, 5]. Figure 1 depicts the structure of etoposide, its administration routes, FDA approval for various cancers, and different strategies for treatment purpose.

Etoposide exerts its cytotoxic effect through the production of double‐strand DNA breaks. Etoposide targets topoisomerase II intracellularly [6]. The DNA and topoisomerase II complex are stabilized by etoposide [7]. Etoposide works by blocking topoisomerase II through the stabilization of the enzyme‐DNA complex, which halts the unwinding of supercoiled DNA, entraps DNA loops, and produces obstruction to DNA activity [8].

**FIGURE 2:**
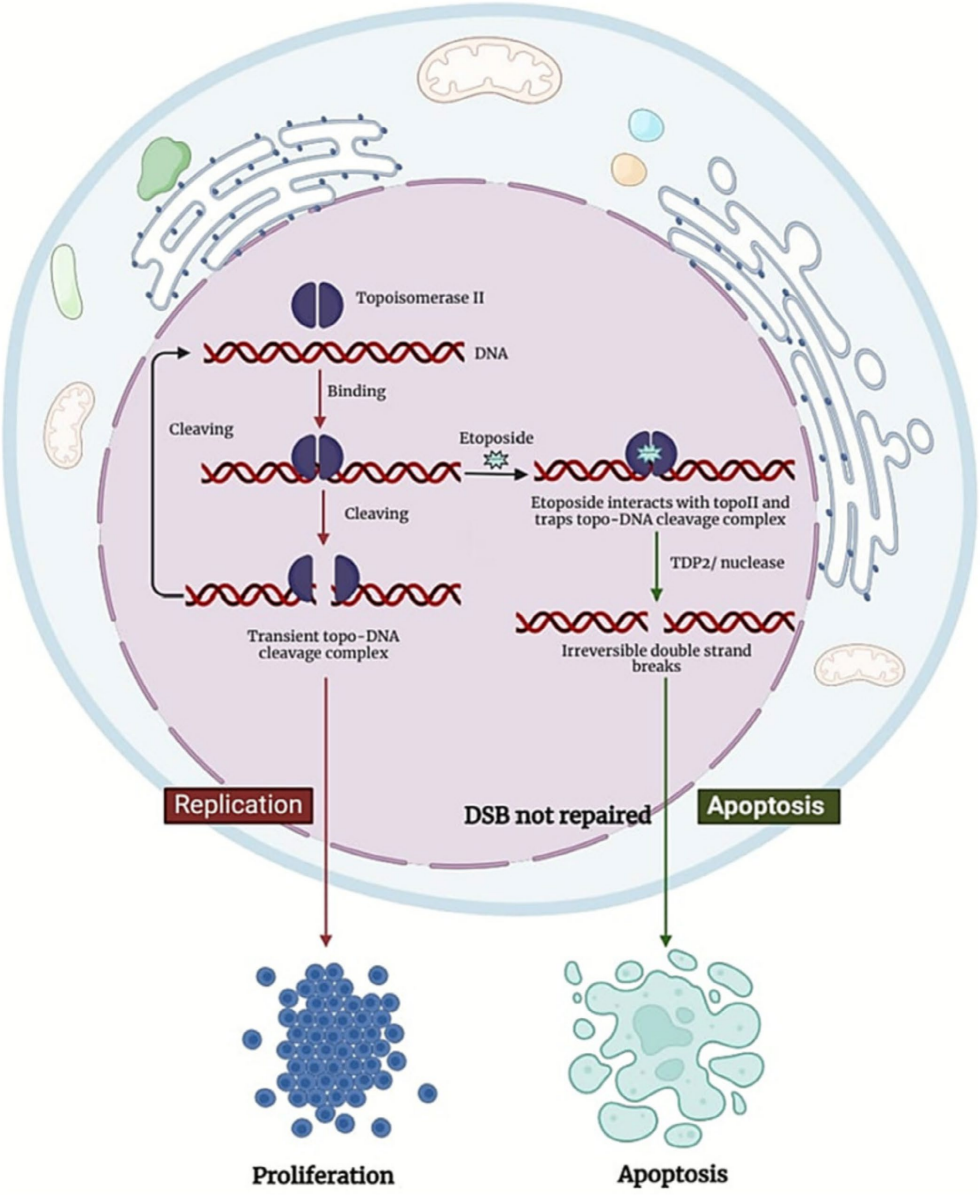
Schematics of etoposide mechanism of action in cancer.

The topoisomerase II enzyme helps in untangling and unknotting DNA by passing single, undamaged DNA via a temporary double‐strand break generated in another helix. Apart from its physiological role, it is also a target for several of the most effective anticancer medications. These drugs increase the amount of covalent cleavage complexes of DNA‐topoisomerase II, which are transitory intermediates in the enzyme's catalytic cycle by stabilizing it and causing many protein‐associated breaks in the DNA. When DNA damage is not properly repaired, apoptosis is triggered and causes cell death [9, 10]. TopoIIα and β are two TopoII isoenzymes that are differentially expressed in mammals during cell growth. The β isoenzyme is present in both proliferating and postmitotic cells, but TopoIIα, a proliferation marker, is abundantly expressed in tumor cells [10]. Topo II inhibitors are classified as poisons or catalytic inhibitors. Poisons stabilize the Topo II–DNA cleavage complex, leading to DNA damage and cell death. Catalytic inhibitors hinder Topo II activity by blocking DNA binding, preventing ATP binding, or stabilizing the non‐covalent Topo II–DNA complex [11]. Topo II poisons are etoposide, teniposide, and the DNA intercalators doxorubicin, daunorubicin, and amsacrine. Topo II catalytic inhibitors are quinolone CP‐115,953, the ellipticines, azatoxins, and the natural flavonoid genistein [12]. Etoposide targets both TopoII isoenzymes; however, it is yet unknown how much TopoIIα and TopoIIβ contribute to the chemotherapeutic effects of etoposide [10]. The FDA‐approved other topoisomerase II inhibitors are teniposide, doxorubicin, idarubicin, epirubicin, and mitoxantrone [13]. Figure 2 illustrates how etoposide functions at the molecular level.

Etoposide, a topoisomerase II inhibitor, has been utilized in various cancer treatments, including cervical cancer, due to its ability to induce DNA breaks. Cervical cancer is a significant health concern worldwide. Cervical cancer is ranked fourth among cancers that impact women worldwide in terms of diagnosis and cancer‐related mortality. The incidence of cervical cancer ranks 5^th^ in transitioned countries and 2^nd^ in transitioning countries [14]. Cervical cancer remains a major concern in transitioning countries may be due to limited awareness, inadequate screening programs, and restricted healthcare access, leading to late‐stage diagnoses and high mortality rates [15]. The incidence of cervical cancer is 11.3 per 100,000 people in transitioned nations, compared to 18.8 per 100,000 people in transitioning countries. Transitioned countries have a mortality rate of 5.2 per 100,000, while transitioning countries face a significantly higher rate of 12.4 per 100,000 [14]. High‐risk human papillomavirus (HPV) vaccination, early diagnosis, and cost‐effective treatment are crucial for reducing this burden. However, low‐resource settings face challenges such as insufficient vaccine infrastructure, inadequate cold chain storage, and economic constraints, resulting in low vaccination rates. Strengthening screening initiatives, improving vaccine accessibility, and developing cost‐effective treatments are essential to lowering cervical cancer incidence in these regions [16].

**FIGURE 3:**
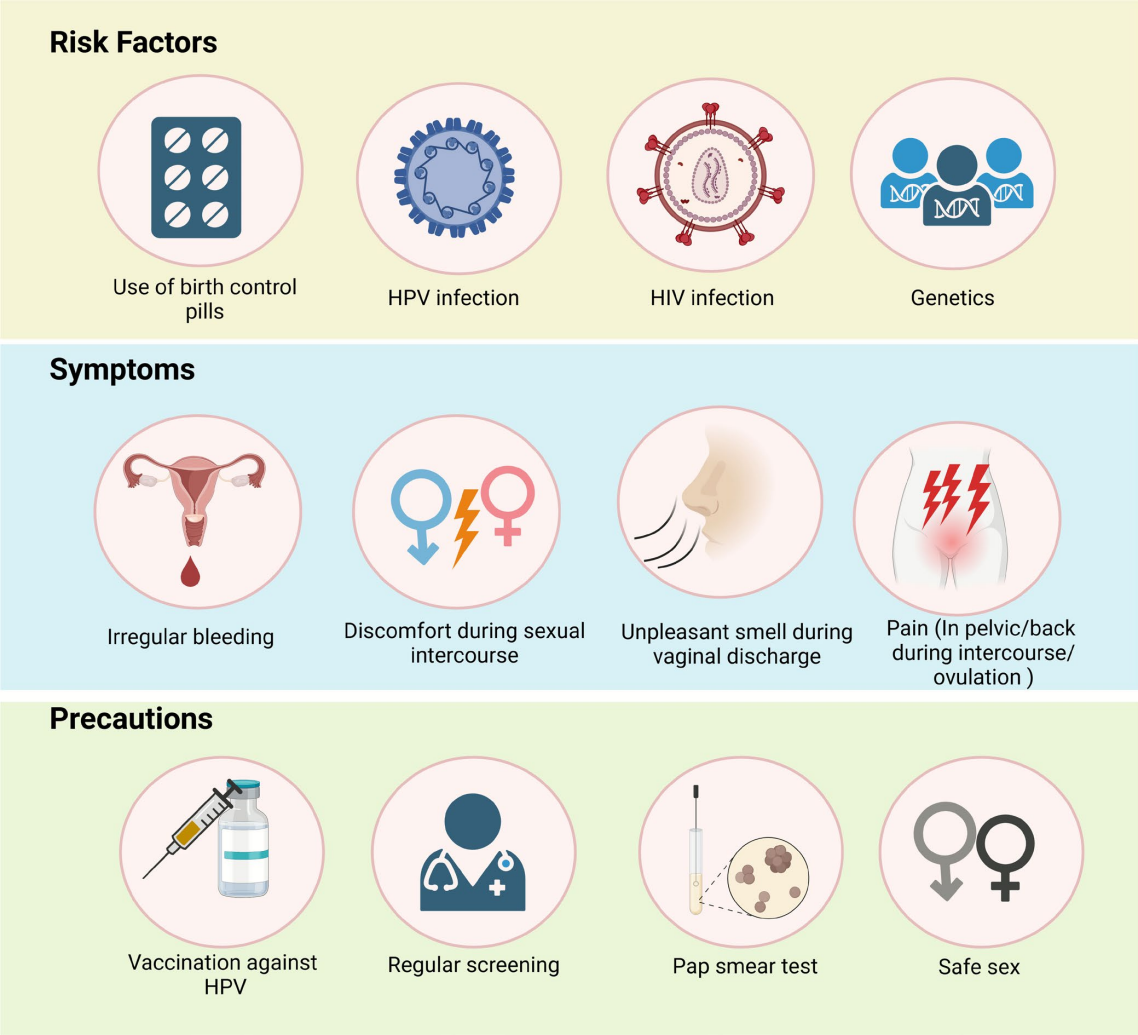
Risk factors, symptoms, and precautions for cervical cancer patients.

HPV infections, especially those caused by HPV 16 and 18, are the primary cause of cervical carcinogenesis [17]. Persistent high‐risk genital HPV infection accounts for about 99.7% of cervical cancer cases [18]. However, not all women with persistent HPV infections will develop cervical cancer, indicating that persistent HPV infections are not a sufficient cause of cervical cancer. *Chlamydia trachomatis* infection and hormonal factors in association with long‐term oral contraceptive usage can be potential cofactors for cervical cancer [17]. Smoking, multiparity, HIV virus infection and AIDS can increase the risk of cervical cancer. Cervical cancer may not exhibit symptoms in its early stages. Unusual vaginal bleeding, discomfort during sexual activity, and foul‐smelling vaginal discharge are the most common signs of cervical cancer. Precautions against cervical cancer include routine screening exams, such as HPV and Pap tests, which help detect precancerous cells and lower the risk of cervical cancer. The HPV vaccine protects against HPV viruses that cause cervical cancer [19]. Figure 3 illustrates the symptoms, treatments, and precautions linked to cervical cancer.

Cervical cancer treatment includes surgery, radiotherapy, chemotherapy, targeted therapy, and immunotherapy, with the choice depending on disease stage and patient factors. Surgery options include conization, trachelectomy, hysterectomy (simple, total, or radical), and pelvic exenteration, often used in early‐stage cases [20]. Patients with tumors less than 2 cm, no lymphovascular space invasion (LVSI), and negative lymph nodes may be eligible for fertility‐preserving treatments such as trachelectomy or conization. Retaining fertility requires careful patient selection and close monitoring. Treatment decisions are based mostly on lymph node assessment, with sentinel lymph node biopsy recommended over pelvic lymphadenectomy due to its lower risk of complications. For individuals at low risk, the SHAPE (Simple Hysterectomy and Pelvic Node Assessment) study suggested simple hysterectomy to reduce surgical morbidity. Less invasive techniques like robotic‐assisted hysterectomy and laparoscopy offer benefits but require specialized expertise. According to the Laparoscopic Approach to Cervical Cancer (LACC) trial, laparoscopic radical hysterectomy may increase recurrence and affect fertility and urinary function [21]. Radiotherapy is a primary treatment modality that includes techniques such as external beam radiation therapy (EBRT), brachytherapy, and intensity‐modulated radiotherapy (IMRT). It is often combined with chemotherapy to enhance therapeutic outcomes. Among these approaches, MRI‐guided adaptive brachytherapy, validated by the EMBRACE‐I trial, has demonstrated superior efficacy. However, since 20%–50% of locally advanced cases may not respond adequately to radiation alone, chemotherapy is frequently incorporated to improve treatment success [22]. Chemotherapy uses drugs to inhibit the growth of cancer cells by either destroying them or preventing their division. It can be administered alone or alongside other treatments to improve effectiveness. The drugs commonly used to treat cervical cancer include cisplatin, carboplatin, gemcitabine, ifosfamide, irinotecan, paclitaxel, topotecan, and vinorelbine [20]. Etoposide is recommended for the treatment of small cell FIGO Stage IA2‐IB2 cervical tumors in combination with cisplatin. This regimen is particularly used for neuroendocrine cervical carcinoma, a rare and aggressive subtype of cervical cancer [23]. Targeted therapies and immunotherapy have significantly improved outcomes for recurrent and metastatic cervical cancer. FDA‐approved pembrolizumab (KEYNOTE‐A18) improved overall and progression‐free survival in PD‐L1‐positive, locally advanced cases, whereas durvalumab (CALLA) showed no significant impact. Tisotumab vedotin (InnovaTV‐301), an antibody‐drug conjugate (ADC) targeting tissue factor, has been approved for recurrent/metastatic cervical cancer. Additionally, HER2‐ and TROP2‐directed ADCs are under investigation [24]. New therapies and combination strategies must be implemented to further improve outcomes. The centralization of care and increased enrollment in clinical trials are essential to advancing treatment options. However, primary and secondary prevention remains the fundamental goal in reducing the burden of cervical cancer [25].

Approximately 75%–80% of invasive cervical cancers are squamous cell carcinomas, and 20%–25% are adenocarcinomas [26]. Compared to squamous cell carcinoma, cervical adenocarcinoma tends to spread to lymph nodes earlier and is less likely to respond to radiation therapy. In particular, when cancer reaches stage II or higher, it can develop outside of the cervix [27]. Neuroendocrine cervical cancer (NECC), a rare and aggressive form of the disease, accounts for 1%–1.5% of cases. For early‐stage NECC, the mainstay of treatment is multimodality therapy with radical surgery along with chemotherapy given as adjuvant or neoadjuvant therapy utilizing etoposide and cisplatin. Locally advanced or recurrent NECC is treated with chemotherapy and radiotherapy. In chemotherapy, etoposide with carboplatin or cisplatin is frequently used [28]. Small cell neuroendocrine cervical cancer (SCNEC) is also a rare type of cervical cancer, constituting 0.9%–1.5% of cases [29]. Compared to squamous cell cervical cancer and cervical adenocarcinoma, SCNEC has a worse prognosis and exhibits more aggressive behavior. Histologically, it is comparable to small‐cell lung cancer [30]. Clinicians treating SCNEC select palliative chemotherapy for metastatic disease, chemo‐radiation therapy for locoregionally progressed disease, and combination modality therapy for limited‐stage disease based on treatment guidelines and retrospective research performed on small cell lung carcinoma [23].

Etoposide induces death in HeLa cells through 2 distinct methods: apoptosis and mitotic death [31]. Etoposide induces apoptosis and G2/M arrest in CaSki cells. Autophagy might occur concurrently with apoptosis [32]. During etoposide‐induced apoptosis in HeLa cells, the activity of Cdk2 kinase (cyclin‐dependent protein kinase 2) increases. Cdk2 kinase activity regulates the mitochondrial translocation of Bax, which controls the loss of mitochondrial transmembrane potential [33].

This article explores the role of oral etoposide, etoposide‐based combination therapies and radiochemotherapy in cervical cancer treatment, focusing on their clinical applications and activity in cervical cancer cells.

## 2 Etoposide Monotherapy

Prolonged oral etoposide (37.5 mg/m^2^, days 1–21 of a 28‐day cycle) was evaluated by Morris et al. [34] for advanced or recurring cervical cancer. Just four of the 44 patients who had received no more than two prior cytotoxic treatment, all of whom had never had chemotherapy, responded. With a median survival of 7.7 months, the median response duration was 2.7 months. Because of low second‐line response rates, etoposide was less successful than conventional palliative regimens, especially in individuals who had already received treatment. It's possible that previous chemotherapy prevented serum drug levels from reaching the 1 μg/mL threshold required for effectiveness. Based on these results, oral etoposide monotherapy may not be very beneficial in this case [35, 36]. In a phase II trial, Rose et al. assessed oral etoposide (40–50 mg/m^2^/day, up to 60 mg/m^2^/day if tolerated) as a second‐line therapy for previously treated squamous cell carcinoma in a phase II trial. Patients had one to eight cycles of chemotherapy. A partial response (40 mg/m^2^/day) and a complete response (30 mg/m^2^/day) were attained by one among the 25 individuals. Prolonged etoposide treatment at this dosage demonstrated minimal effectiveness as a second‐line therapy [37]. Extended oral etoposide was studied by Rose et al. in cervical nonsquamous cell cancer. Patients who had never undergone chemotherapy were given 50 mg/m^2^/day for 21 days every 4 weeks (or 40 mg/m^2^/day if they had previously received radiation), with dosage increases if tolerated. With an average of three treatments given to patients, the response rate was 11.9% (7.1% complete, 4.8% partial). Respondents had a median response length of 4.7 months and had never received chemotherapy before. Etoposide was less effective than second‐line chemotherapy, while being moderately beneficial in individuals who never underwent chemotherapy [38]. In a trial, 15 patients with recurrent or persistent cervical cancer after chemotherapy and radiation therapy were given continuous low‐dose oral etoposide (50 mg/day, 14 days per 21‐day cycle). Six patients had progressing illness, one had stable disease, and two (16.6%) achieved a partial response. Although etoposide was less tolerable than cyclophosphamide, there was no significant difference in the overall response. Following chemoradiation, it may be taken into consideration for the treatment of persistent or recurrent cervical cancer [39].

## 3 Combination Therapy

**FIGURE 4:**
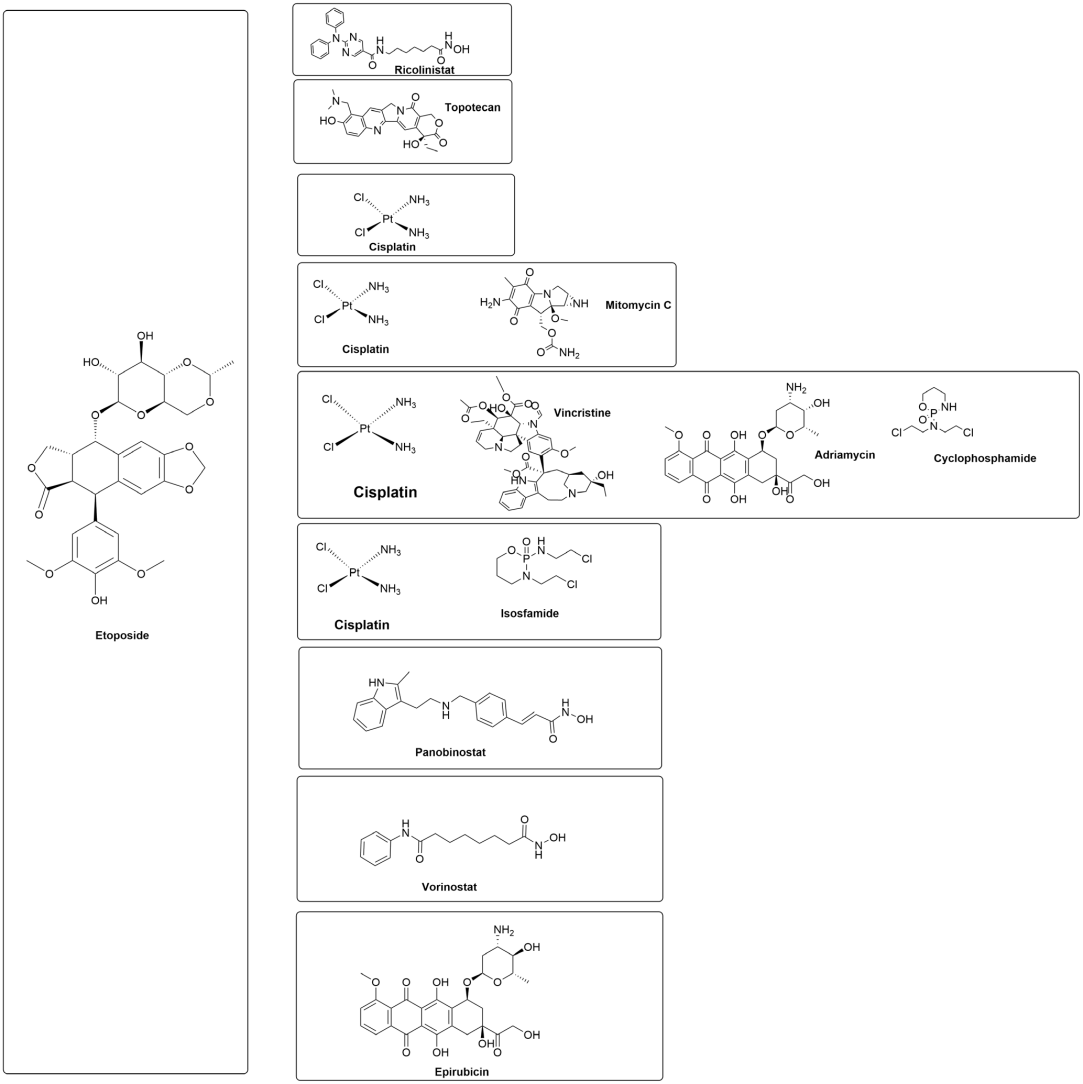
Different combination therapies using etoposide.

The one‐dimensional mechanism of action of single‐agent therapy frequently enhances and activates alternate pathways, which encourages the establishment of mutations that confer chemoresistance and tumor relapse [40, 41]. Single‐agent treatment regimens for cancer patients frequently result in unfavorable side effects, drug resistance, decreased intake, etc. Contrary to single‐drug treatment, combination therapy can control different signaling pathways, which increases cytotoxicity but with less of each drug and may even overcome resistance mechanisms. Therefore, combining two or more drugs is an essential strategy [42]. Combination medicines are therefore employed to improve target selectivity and stop the emergence of drug resistance [40, 41]. Notable combination approaches include etoposide and HDAC inhibitors, which enhance cancer cell susceptibility by promoting chromatin relaxation; etoposide and topotecan, which target both Topo I and Topo II to reduce drug resistance; and etoposide, cisplatin, and other agents, which act through complementary mechanisms to increase DNA damage and apoptosis, improving therapeutic outcomes. The Figure 4 illustrates the utilization of etoposide in combination with various chemotherapeutic agents.

### 3.1 Etoposide and HDAC Inhibitors

Epigenetics plays a significant role in cancer, and HDACs are significant modulators of gene expression and cell cycle control. In addition to their anticancer effects, HDAC inhibitors increase the efficacy of other therapies. Preclinical research indicates that pretreatment with low‐dose HDAC inhibitors increases the susceptibility of cancer cells to Topo II toxins, such as etoposide, by causing chromatin relaxation and downregulating DNA repair proteins. Both in vitro and in vivo, this combination has potential as a therapy by inducing apoptosis and DNA damage [43].

Kaur et al. studied the combined effects of topoisomerase inhibitors and ricolinostat. The Chou and Talalay equation's combination index (CI) value was used to identify interactions between combinations of ricolinostat and topotecan or etoposide. The indicators for additive, antagonistic, and synergistic effects are CI = 1, CI > 1, and CI < 1, in that order. For a duration of 72 h, etoposide and ricolinostat were administered in combination to HeLa and SiHa cells at dose ratios of 0.25, 0.5, 1, 1.25, and 1.5 times their individual IC_50_ values. In SiHa and HeLa cells, the etoposide IC50 values were 1.399 and 9 μM, respectively, during a 72‐h treatment period. Both cell lines' viability was considerably reduced by the tested combination doses as compared to single medications. In the case of HeLa cells, the inhibitor interactions were synergistic for all the doses examined. In SiHa cells, all inhibitor doses tested in combination were synergistic, except for one, which was found to be additive. The HeLa cell line exhibited synergistic effects with the lowest CI (0.38460) when treated with ricolinostat and etoposide at 0.25 times the IC50, while the SiHa cell line exhibited the lowest CI (0.27523) when treated with 1.5 times the IC50. These findings suggested that ricolinostat plus etoposide was the most effective combination in HeLa cells [44].

Data from both preclinical and clinical studies have demonstrated how DNA topoisomerase inhibitors and HDAC inhibitors function synergistically. In this study, the use of the topoisomerase II inhibitor etoposide in combination with the histone deacetylase inhibitor vorinostat was investigated in HeLa cells. Both vorinostat and etoposide reduced HeLa cell viability when used individually in a dose‐dependent manner. Serial dilutions of 50% inhibitory concentration (IC50) (72 h) of free vorinostat or etoposide were given to HeLa cells at a constant ratio of 1:0.3 to determine the combination index (CI). The amount of growth inhibition was measured 72 h after exposure. Vorinostat and etoposide combined therapy produced a synergistic effect with a small CI (CI ≤ 0.4) [42].

Wasim and Chopra investigated the effects of etoposide in combination with panobinostat, a pan histone deacetylase inhibitor, on HeLa and SiHa cervical cancer cells. The cells were treated with serial dilutions of each medication, either singly or in combination, at fixed dose ratios of 0.25, 0.5, 1, 1.25, and 1.5 times the corresponding 72‐h IC50 values. Three distinct sequences of analysis were conducted to study the combined drug effects. In the first sequence, etoposide and panobinostat were administered simultaneously; in the second sequence, panobinostat was introduced first; and in the third sequence, etoposide was introduced first. A low CI (CI < 0.3) was achieved with a high synergistic impact when panobinostat and etoposide were given to both cell lines simultaneously. A minor synergistic effect was observed when the cells were pretreated with etoposide for 24 h before panobinostat was added. Adding panobinostat before etoposide resulted in antagonistic effects on HeLa cells but synergistic effects on SiHa cells. These results suggest a schedule‐dependent synergistic interaction between panobinostat and etoposide. The IC50 of panobinostat at 72 h decreased in HeLa cells from 150 to 25.4 nM (DRI 5.9), and in SiHa cells, the IC50 decreased from 50 to 2.63 nM (DRI 19) when Panobinostat was combined with etoposide. Panobinostat and etoposide combined therapy significantly increased the activation of caspase 3/7 in SiHa and HeLa cells compared to that in control cells and etoposide or panobinostat treatment alone [45].

### 3.2 Etoposide and Topotecan

As Topo I and Topo II enzymes have overlapping roles in DNA metabolism, their combined inhibition enhances anticancer activity. Drug resistance can also be decreased by this double targeting since downregulating one Topo frequently results in the overexpression of the other. To overcome resistance, this combination is an effective strategy [46].

Boabang et al. used the squamous cell cervical cancer cell lines CaSki, C‐33 and A‐431 to evaluate the cytotoxic effect of topotecan individually and in combination with etoposide. Topotecan was more effective than etoposide in CaSki cells. Topotecan increased the cytotoxic effect of etoposide in all three cell lines. However, a synergistic effect was observed only in A‐431 cells. When used alone, etoposide showed strong cytotoxicity in C‐33 and CaSki cells and less cytotoxicity in A‐431 cells. The combined effect of both drugs might be due to their synergistic inhibitory effect on DNA repair. It is known that topoisomerase II levels increase when topoisomerase I is inhibited. Cancer cells are more susceptible to topoisomerase II inhibitors, such as etoposide, when topoisomerase II levels increase. In this way, topotecan may increase the efficacy of etoposide. The combination may aid in treating squamous cell cervical cancer and carcinoma of the vulva if these findings are validated with native squamous cell carcinomas and medication sequences are discovered [47].

### 3.3 Etoposide, Cisplatin and Others

Etoposide and cisplatin are widely used chemotherapeutic agents that target DNA integrity through distinct but complementary mechanisms. Cisplatin primarily induces DNA damage by forming platinum‐DNA adducts, triggering apoptosis through oxidative stress, calcium signaling disruption, and mitochondrial impairment. Its effects are modulated by key signaling pathways, including MAPK, JNK, p38, and Akt, while resistance mechanisms involve alterations in apoptotic pathways, oxidative stress adaptation, and transporter regulation [48]. Etoposide effectively stabilizes the Topo II–DNA cleavage complex, which stops DNA from re‐ligating and forms a barrier that causes DNA breaks during transcription and replication [8]. These medications work together to increase DNA damage, overpowering the cell's defenses and encouraging the death of cancer cells, which is why they are useful in chemotherapy treatments. For cervical cancer therapy, these drugs are often combined with agents like mitomycin, paclitaxel, epirubicin, carboplatin, and vincristine to enhance treatment efficacy. Radiotherapy is also commonly used alongside these regimens, targeting multiple tumor progression pathways for improved therapeutic outcomes.

Gil‐Ibañez et al. [49] reported a case of a 34‐year‐old pregnant woman treated with neoadjuvant chemotherapy while preserving pregnancy. The woman was diagnosed with stage IIA1 large‐cell NECC at 21 weeks of gestation. The chemotherapy regimen included three cycles of etoposide (100 mg/m^2^) and cisplatin (50 mg/m^2^) every 3 weeks. There was no toxicity associated with the treatment, and foetal development was good. At 31.4 weeks gestation, she underwent a cesarean section, and radical hysterectomy was simultaneously performed. The patient also received adjuvant chemoradiotherapy (radiotherapy and five cycles of concomitant weekly cisplatin at 40 mg/m^2^). The patient showed no evidence of recurrence or metastasis, and the baby had no neurodevelopmental disorders at 38 months after the operation.

A case report by Bajaj et al. described a patient with stage IVB SCCC who was treated with concurrent chemotherapy and radiation therapy (CCRT) and EP chemotherapy. Radiation and chemotherapy were started simultaneously, and chemotherapy was continued adjuvantly for a total of six cycles. The chemotherapy regimen included intravenous administration of cisplatin (50 mg/m^2^) on the first day and etoposide (100 mg/m^2^) on the first 3 days of every 3‐week cycle. The patient was followed for 6 years and was without any evidence of disease at that time [50].

Pei et al. investigated SCNEC patient outcomes and prognostic markers, as well as the impact of adjuvant therapy on patient survival following radical surgery for patients with FIGO stages I‐II. This study determined the optimal number of cycles of EP. The EP regimen consisted of intravenous administration of etoposide (100 mg) and cisplatin (30 mg) on days 1–3 for one to eight cycles. Of the 16 patients treated with EP for one to three cycles, 11 experienced disease recurrence within 29 months. Compared to patients receiving < 4 cycles (RFS = 34.7%) or no systemic chemotherapy (RFS = 0%), patients receiving ≥ 4 cycles of EP had a better 5‐year RFS (recurrence‐free survival 54.8%). Those treated with ≥ 5 cycles of EP had a 5‐year RFS of 67.6%, and those treated with ≥ 6 cycles of EP had a 5‐year RFS of 67.9%. Compared to other patients, those who underwent adjuvant chemotherapy and received at least five cycles of EP had a better prognosis [30].

In a case report by BacalbaȘa et al. [51] a 41‐year‐old individual with large cell neuroendocrine cervical carcinoma (NECC) was treated with cisplatin and etoposide as adjuvant therapy along with radical surgery. On days 1–3 of the 4‐week course, etoposide (100 mg/m^2^/day) was administered, and on day 1 of the 4‐week course, 60 mg/m^2^ cisplatin was administered. Six rounds of chemotherapy were given in total. The patient had neither distant nor local recurrence during the 1‐year follow‐up.

Wang et al. [52] described a case of an 18‐year‐old female patient who received radiation and chemotherapy for cervical small cell carcinoma. The patient was diagnosed with stage IB2 SCCC 47 days after delivery. The patient underwent radiotherapy after neoadjuvant chemotherapy (NACT). Approximately 9 weeks of intravenous cisplatin (70 mg/m^2^) on days 1–3 together with etoposide (70 mg/m^2^) on days 1–5 were used as the chemotherapy regimen. After four cycles of NACT, the tumor size decreased by 90%, and the patient received radiation therapy 3 weeks after the completion of NACT. An additional four cycles of chemotherapy were administered when the patient showed no evidence of relapse. When the case was reported, the patient had been in clinical remission for 8 months following treatment.

In one study, 30 patients with recurrent cervical carcinoma were given oral etoposide (25 mg/day) for 21 days, and cisplatin (50 mg/m^2^) was given intravenously on day 1 of every week. Of the 30 patients, 27 had prior therapy. After confirming safety, the dose of etoposide was increased to 50 mg/day. The overall response rate among the 25 evaluable patients was 16.7%. The OS duration was 9.7 months, with an average PFS of 4.5 months. The hematologic toxicities included grade 3 or 4 leukopenia (63.3%), neutropenia (58.6%), anemia (50.0%), and thrombocytopenia (20.0%), and four patients developed febrile neutropenia. Nonhematologic toxicities included vomiting (6.7%), grade 3 nausea, anorexia (13.3%), and fatigue (6.7%). According to the authors' findings, the combination of oral etoposide and intravenous cisplatin is safe and effective for use in recurrent cervical cancer [53].

Bae et al. studied the effectiveness of cisplatin and etoposide‐based neoadjuvant treatment. Ninety‐nine individuals with cervical cancer, which was locally advanced (stages 1B–2B), underwent radical hysterectomy after receiving neoadjuvant chemotherapy (NACT), which included cisplatin and etoposide. Every 10 days, patients were given three courses of NACT intravenously. Etoposide (100 mg/m^2^) was given on day 1, and cisplatin (60 mg/m^2^) was given on days 1 and 2. Within 2–3 weeks of NACT, a type III radical hysterectomy was performed. The 3‐year progression‐free survival (PFS) was 77.2%, and the overall survival (OS) was 91.3%. The five‐year OS was 88.1%, and the PFS was 60.5%. This study showed promising results (i.e., better survival outcomes and moderate and reversible toxicity), and the course of cisplatin and etoposide is relatively less expensive than other regimens [54].

In a study by Al‐Saleh et al. 38 women with primarily recurrent or advanced cervical cancer received 3 days of intravenous etoposide (60 mg/m^2^/day) and cisplatin (30 mg/m^2^/day), followed by 7 days of oral etoposide (50 mg), which was repeated every 28 days. With 7 patients demonstrating a CR and 8 demonstrating a PR, the response rate was 39% (15 patients). The response duration ranged from 2 to 36 months, with a 1‐year survival rate of 65% and a 2‐year survival rate of 37% among 15 patients. In contrast, non‐responders had a 1‐year survival of 6%, and there were no 2‐year survivors. The response rate for recurrent disease was 50% (65% in nonirradiated areas and 42% in previously irradiated areas), and the response rate was 10% for primarily advanced disease. The disease progressed in 16 patients, while seven patients had stable disease. Symptoms improved in 23 patients, remained unchanged in seven patients, resolved in five patients, and worsened in three patients. The response rate and duration of cisplatin analogue treatment are inferior to those of etoposide and cisplatin combination treatment when used as a single agent. Etoposide and cisplatin treatment is neither more nor less efficient than other cisplatin‐based combinations against advanced or recurring cervical cancer. In this study, the sequences used were etoposide and cisplatin [47]. Correctly placing the two medications in the dosage regimen might increase the treatment efficacy. Other studies have shown that using these drugs in reverse order has less hematological toxicity and is more effective [48]. The comparatively low toxicity of this regimen in the palliative scenario could be a benefit [55].

In a study by Hoskins et al. four cycles of etoposide and cisplatin were given to 11 patients with small cell cervical cancer. Each cycle lasted for 5 days, with an interval of 14 days between cycles. Etoposide (40 mg/m^2^) followed by cisplatin (25 mg/m^2^) was given on each day of treatment. Starting with the subsequent cycle of chemotherapy, radiotherapy was given concurrently. Every patient had no prior experience with radiation or chemotherapy. Two of the eight evaluable patients experienced a partial response, whereas five of the patients experienced a complete response. Among the 11 patients, 1 was the primary progressor, and among the remaining non‐progressors, 4 experienced relapses, and 2 died because of toxicity. The study's findings indicate that this treatment is suitable for women whose entire evident tumor is contained in a radiation field and whose performance status is either zero or one [56].

In order to evaluate the safety and efficacy of mitomycin C, etoposide, cisplatin, and epirubicin (MEPA) as neoadjuvant treatment in patients with cervical cancer, Obata et al. undertook research. Fourteen individuals with cervical cancer received neoadjuvant MEPA therapy before undergoing radical hysterectomy. Every 4 weeks, the MEPA regimen was repeated and comprised the following dosages: Mitomycin C (15 mg/m^2^) on day 1, etoposide (70 mg/m^2^) on days 1 through 3, cisplatin (15 mg/m^2^) on days 1 through 5, and epirubicin (30 mg/m^2^) on day 1. Each patient underwent radical hysterectomy following two or three sessions of chemotherapy. Of the 14 patients, seven achieved complete remission (50%), and six achieved partial remission (43%). Only 1 patient showed no change (7%). Overall, 93% of the patients achieved a clinical response. Two patients had microscopic residual disease (less than 5 mm), and 6 individuals had no residual disease, as determined by an examination of the surgical material. Compared to patients with macroscopic residual disease, those with no residual disease or microscopic disease had a noticeably longer life. Myelosuppression was found to be the dose‐limiting toxicity. A good rate of complete response was observed with MEPA therapy, despite severe myelosuppression [57].

In a case report by Nagai et al. [58] a 28‐year‐old woman with stage 2B cervical cancer was treated with neoadjuvant intraarterial chemotherapy (NAC). The woman was diagnosed at 23.5 gestational weeks and underwent elective cesarean section after 29 gestational weeks. Three days after the operation, three cycles (each lasting 21 days) of chemotherapy containing etoposide (350 mg/m^2^), carboplatin (500 mg/body, AUC 4.5), and epirubicin (40 mg/m^2^) were administered. Chemotherapy markedly reduced the tumor size, and then extended radical hysterectomy was performed. The patient was still alive at the reporting time, 6 years after treatment.

Hara et al. [59] used five distinct cervical cancer cell lines—OMC‐4, HeLa, TMCC‐1, CAC‐1, and JSK/CA‐1 to investigate the growth inhibitory properties of 15 standard antitumor drugs, including etoposide. Drugs were used at four different concentrations: 0.01X, 0.1X, 1X, and 10X peak plasma concentrations (PPCs according to data supplied by pharmaceutical companies). The IC50 values of etoposide for CAC‐1, HeLa, TMCC‐1, and JSK/CA‐1 cells were less than 0.1X that of PPC, but for OMC‐4 cells, the value was greater than 0.1X that of PPC. The five antitumor drugs etoposide, epirubicin, mitomycin C, vinblastine, and adriamycin, which showed increased antitumor activity in many cell lines, were used for combination studies of these 2 drugs. Mitomycin C and etoposide were the most effective combination in TMCC‐1 cells and the second‐most effective combination in OMC‐4 cells. Etoposide and epirubicin were the most effective combination in HeLa cells and the third most effective in TMCC‐1 and JSK/CA‐1 cells. Etoposide and adriamycin were the second‐most effective combination in JSK/CA‐1 and HeLa cells. Etoposide and vinblastine were the most effective combination in JSK/CA‐1 cells. Based on the total number of appearances in the best 3 combinations in all cell lines, etoposide (8 appearances) and mitomycin‐C (7 appearances) were considered better than the other drugs. All 5 drugs were also used in combination with IFN‐γ, and etoposide in combination with IFN‐γ was the most effective.

Morris et al. [60] conducted a pilot study involving 10 patients with small‐cell cervical cancer who were treated with combination chemotherapy regimens that included doxorubicin, etoposide, and cisplatin. Combination chemotherapy was given as adjuvant therapy. Of the 10 patients, 7 had stage IB cancer, 1 had stage IIA cancer, and 2 had stage IIB cancer. Etoposide (75 mg/m^2^) was administered on days 1–3, and cisplatin (50 mg/m^2^) and doxorubicin (50 mg/m^2^) were administered on day 1 and every 28 days (2–6 cycles). Of the seven patients that had been evaluated, one had a partial reaction and three had a complete response; this means that the response rate was 57%. One patient had a progressing illness, and the other two had stable conditions. The authors concluded that this chemotherapy regimen had shown activity in small‐cell cervical carcinoma, and further studies should be performed to evaluate its use in individuals with early‐stage illness as an adjuvant to surgery or radiation. It might also increase disease‐free survival.

Sixteen patients with cervical adenocarcinoma or adenosquamous carcinoma who had not undergone previous therapy were treated with 3–5 sessions of neoadjuvant chemotherapy using cisplatin, mitomycin C, and etoposide in a study by Iwasaka et al. [27] Twelve patients, either in stage I or II, had radical hysterectomy after chemotherapy; three, who had positive pelvic nodes, had adjuvant radiotherapy; three, in stages IIB, IIIB, and IVB, had standard radiation therapy; and one, in stage IIIB, had chemotherapy using a different regimen since MEP therapy was ineffective. Every 4 weeks, patients received the following treatments: on day 1, etoposide (100 mg/m^2^), cisplatin (50 mg/m^2^), and mitomycin C (10 mg/m^2^). On days 3 and 5, patients also received etoposide. 50% response rate was obtained from 3 complete replies and 5 partial responses. There was no discernible difference in the two groups' mean survival times (47.5 months for responders and 28.3 months for non‐responders). The authors suggested that the MEP chemotherapy regimen may be effective for treating cervical adenocarcinoma.

In a study conducted by Umesaki et al. [61] etoposide, mitomycin C, and cisplatin were used to treat 31 individuals with recurrent cervical adenocarcinoma or stage IVB cancer by the Japanese Gynecologic Oncology and Chemotherapy Study Group. On day one, 50 mg/m^2^ cisplatin and 10 mg/m^2^ mitomycin C were administered intravenously, and on days 1, 3, 5, 100 mg/m^2^ etoposide was given intravenously. Among 25 patients with recurrent adenocarcinoma, one achieved a partial response (PR), and four achieved a complete response (CR). Patients who responded had an average of 4–5 MEP courses. Two of the patients who responded to therapy survived for more than 3 years. MEP therapy had a response rate of 16.1%, while cisplatin alone had a response rate of 20%. MEP therapy is therefore not better than single‐agent cisplatin therapy. However, the MEP response rate was 26.7% in patients who had not received chemotherapy. This finding suggested that MEP therapy worked well for individuals who had not previously received chemotherapy.

Phase II study using etoposide, ifosfamide (and mesna), and cisplatin was carried out in 14 patients with advanced and recurrent cervical cancer who were no longer receptive to surgery or radiation therapy. on a maximum of six cycles, intravenous administration of etoposide (75 mg/m^2^), cisplatin (25 mg/m^2^), and ifosfamide (1.0 g/m^2^) was administered on 3 days in a row every 28 days. Eight (57%) of the thirteen patients who qualified for assessment had a full reaction, and none had a partial one. Out of the eight responders, three had no illness, and five had relapsed 10–18 months after their first response. This combination shown to be safe and tolerable when given to individuals with advanced or recurrent cervical cancer. The results of this pilot research are positive, despite the short follow‐up period (mean follow‐up of 14 months) [62].

Wu et al. [63] described a case in which neoadjuvant chemotherapy was used for fertility‐sparing treatment. A 25‐year‐old woman was diagnosed with exophytic cervical small cell neuroendocrine carcinoma (SCNEC). She was treated with neoadjuvant chemotherapy (NACT). Preoperative chemotherapy consisted of two cycles of ifosfamide, etoposide, and cisplatin, and radical abdominal trachelectomy surgery was performed. To reduce the risk of oocyte loss, postoperative chemotherapy included six cycles of IEP in addition to gonadotropin‐releasing hormone agonist (GnRHa). She gave birth to a baby 7 years after treatment through cesarean section.

In a study by Chang et al., radical hysterectomy and adjuvant chemotherapy were given as the primary therapy to 23 patients with stage IB‐2 small‐cell cervical cancer. Of the 23 patients, 14 received VAC/PE (VAC alternating with PE), 8 received PVB (cisplatin, vinblastine, and bleomycin), and 1 received a different regimen. The VAC/PE regimen involves intravenous administration of vincristine (1.0 mg/m^2^), doxorubicin (40 mg/m^2^), and cyclophosphamide (1000 mg/m^2^) on day 1 and infusion of cisplatin (100 mg/m^2^) on day 1 and etoposide (100 mg/m^2^) on days 1–3. Treatment was given within a month of radical hysterectomy and repeated every 21 days (for six courses). For the meta‐analysis, a total of 17 individuals with small‐cell cervical cancer who received adjuvant chemotherapy following a hysterectomy, as documented in the English literature, were pooled into this study (for a total of 40 patients). A variety of chemotherapy regimens were used in the studies that were published in the literature. Therefore, the chemotherapies were divided into two groups. Treatments similar to those used for small cell lung cancer were assigned to the first group of 28 patients (VAC/PE regimens), while other treatments were assigned to the second group of 12 patients. Compared to 33% of patients who had regimens other than VAC/PE, 68% of individuals who received regimens similar to those used to treat small cell lung cancer were still alive at the time of their last follow‐up. These results suggest that after radical hysterectomy, chemotherapy regimens such as those used to treat small cell lung carcinoma might be favorable for treating small cell cervical cancer [64].

A study by Boruta et al. [65] included 34 patients who had been diagnosed with early‐stage NECC and had undergone chemotherapy and radial surgery either in combination or separately with radiation therapy. To determine prognostic factors and select an appropriate multimodality therapy, a meta‐analysis of the literature was conducted. Postoperative vincristine, adriamycin, and cyclophosphamide (VAC) to seven, platinum and etoposide (PE) were administered to fifteen patients, PE and VAC were administered in alternate cycles to two patients, and other chemotherapy regimens were administered to 10 patients. PE and VAC chemotherapy were linked to better survival than the other regimens. There was no difference between the groups treated with PE or VAC. However, PE‐containing regimens are usually preferred over VAC because they are less toxic.

Tokunaga et al. [66] studied nine patients with small cell cervical cancer who were treated with concurrent chemoradiotherapy (CCRT). Five patients had stage IB disease and underwent CCRT after radical hysterectomy, and 4 had advanced‐stage disease and primarily underwent CCRT. Within a week after starting radiation therapy, three cycles of concurrent chemotherapy were administered every 6 weeks. Intravenous infusions of vincristine (1 mg/m^2^), adriamycin (40 mg/m^2^) and cyclophosphamide (1000 mg/m^2^) were given on day 1, cisplatin (100 mg/m^2^) was given on day 22, and etoposide (100 mg/m^2^) was given on days 22–24. CCRT had a 75% response rate when used as a primary therapy. At 5 years, all patients had OS and PFS rates of 52% and 56%, respectively. Three‐year overall survival (OS) was 25% for individuals with advanced‐stage cancer and 80% for those with early‐stage illness. The two groups did not, however, differ greatly from one another. The study's results indicate that, in addition to surgical removal, CCRT treatment with a VAC/PE regimen in individuals with early‐stage cancer may improve local control and survival.

Thirty‐one of the 34 patients with small cell cervical cancer (SCCC) in an additional study by Hoskins et al. [67] received combined modality treatment, which included combination chemotherapy based on platinum and radiation. Two protocols, SMCC and SMCC2, were used to treat SCCC, and both included the use of etoposide and cisplatin and field irradiation along with concurrent chemotherapy. In SMCC2 cells, in addition to carboplatin, paclitaxel, and para‐aortic irradiation were used. Seventeen of the 31 patients were treated with SMCC (the SMCC protocol is discussed in the above study), and 14 were treated with SMCC2. In SMCC2 cells, paclitaxel (175 mg/m^2^) was given on day 1, followed by cisplatin (60 mg/m^2^), which was given on days 1 and 2. In the next cycle, cisplatin (60 mg/m^2^/day) followed by etoposide (75 mg/m^2^/day) was administered on days 21 and 22. Oral etoposide (100 mg/day) was subsequently administered to patients on days 23, 24, and 25. During locoregional irradiation (beginning at the same time as the third chemotherapy cycle), cisplatin was given at biweekly intervals on days 42, 56, 70, and 84. On day 98, the patient received two additional cycles of chemotherapy consisting of carboplatin (5 or 6 area under the curve) and paclitaxel (175 mg/m^2^). On day 126 of the last cycle, carboplatin was administered with oral etoposide for 5 days. Thirty‐two percent of patients either progressed or relapsed after treatment. For 31 patients, the 3‐year overall survival (OS) was 60%. The failure‐free survival (FFS) rate for 31 patients was 57%. After 3 years, the survival curve plateaued, suggesting that these patients are likely to have healed. Local failure occurred in 13% of patients, while distant failure accounted for 28% of all failures. Approximately 55% of individuals with SCCC can be successfully treated with a combination of chemotherapy and radiation. The results of the two protocols did not significantly differ, and they were said to be better than any other results previously published in the literature. They suggested that regimens routinely using chemotherapy as well as local radiation treatment yield better results than regimens that do not use chemotherapy. SMCC2 is more toxic haematologically, and the duration of SMCC2 treatment is longer than that of the SMCC protocol. With SMCC2, the distant relapse rate decreased, and the tolerability of the regimen improved.

Zivanovic et al. conducted a retrospective analysis using the Virginia K. Pierce database. Seventeen patients with small cell neuroendocrine cervical carcinoma (SCNEC) were analyzed. Two patients received chemoradiation therapy, and 5 received chemotherapy as primary therapy. The chemotherapy regimen included intravenous administration of cisplatin (60 mg/m^2^) on day 1 and etoposide (100 mg/m^2^) daily for 3 days every 3 weeks (4 courses) concurrently with radiotherapy. The chemotherapy regimens included carboplatin (area under the curve 4–5) and etoposide (100 mg/m^2^), which were administered every 3–4 weeks. Of the 17 patients, 11 had FIGO stage IA2‐IB2 disease (early‐stage disease), of which 6 received chemotherapy and 5 did not. All five patients developed recurrent disease and distant metastases. Of the 6 patients who received chemotherapy, only 2 developed recurrent disease, and 1 developed distant metastasis. Patients who received chemotherapy had greater survival rates as compared to those who did not. Based on these findings, the authors proposed that systemic platinum and etoposide‐based chemotherapy might prevent patients with early‐stage cancer from developing distant metastases [23].

A total of 179 patients with small cell neuroendocrine cervical carcinoma (SCNEC) FIGO stages I–IV were analyzed in a retrospective study. Among the 56 patients with FIGO stage IIB‐IVB disease, 16 patients treated with at least five cycles of etoposide and platinum as primary therapy had significantly superior 5‐year failure‐free survival = 42.9% (FFS) and CSS (cancer‐specific survival = 45.6%) compared to the other 40 patients who received different primary treatments (FFS = 11.8%, CSS = 17.1%). Additionally, in eight patients, simultaneous chemoradiation with at least five cycles of etoposide and platinum was linked to considerably improved 5‐year FFS (62.5%) and CSS (75.0%) compared to those in 48 patients receiving other treatments (FFS = 13.1%, CSS = 16.9%). In stages IIB–IVB and earlier stages, CCRT with five or more cycles of etoposide and cisplatin (EP) might be the treatment of choice [68].

Salvo et al. [69] selected 453 patients who were diagnosed with neuroendocrine cervical carcinoma between 1986 and 2022 from the Neuroendocrine Cervical Tumor Registry for retrospective analysis. Across all stage categories, the most common primary chemotherapy regimens were cisplatin/carboplatin and etoposide. A total of 96/106 patients in the early stage (91%), 137/156 patients in the locally advanced stage (88%), and 70/84 patients in the advanced stage (83%) received cisplatin/carboplatin and etoposide as their primary chemotherapy treatment. Treatment with cisplatin, carboplatin, and etoposide was a positive predictor of overall survival in patients with advanced disease (hazard ratio = 0.33).

A case report by Balderston et al. [70] reported the long‐term survival of female patients receiving etoposide combination therapy. A 22‐year‐old woman who was 30 weeks pregnant with twins was diagnosed with stage IIA small cell cervical cancer. Pelvic irradiation and multiagent chemotherapy were used in the patient's treatment. The patient had two cycles of VAC (vincristine 1.2 mg/m^2^/week for 3 weeks, dactinomycin 300 μg/m^2^/day for 5 days, and cyclophosphamide 150 mg/m^2^/day for 5 days) in addition to three rounds of etoposide (400 mg/m^2^) and cisplatin (80 mg/m^2^). Radiotherapy was subsequently administered. An additional four courses of cisplatin and etoposide were given when she had no evidence of disease. The authors reported long‐term survival of up to 5.5 years for patients without any evidence of the disease. Table 1 presents a compilation of research findings regarding the application of etoposide chemotherapy in combination with cisplatin, doxorubicin, paclitaxel, vincristine, cyclophosphamide, and mitomycin C in individuals with various types of cervical cancer.

**TABLE 1:** Research findings on etoposide chemotherapy combined with various agents in cervical cancer.

Study	Number of evaluable patients	Prior therapy	Etoposide dosage	Concurrent therapy	Complete response (CR)	Partial response (PR)	Stable disease (SD) and progressive disease (PD)	Progression‐free survival (PFS) or failure‐free survival (FFS)	Overall survival (OS)
Umesaki et al. (1999)	31	No prior chemotherapy 15 (52%) Chemotherapy 16 (48%)	100 mg/m^2^ of etoposide on days 1, 3 and 5	On day 1 50 mg/m^2^ of cisplatin 10 mg/m^2^ of MMC	4 CR (12.9%)	1 PR (3.2%)	9 SD 17 PD	—	8.7 months (range 0.4–59.2 months)
Morris et al. (1992)	7	Radical hysterectomy (3) radiotherapy (1)	75 mg/m^2^ etoposide on days 1–3 repeated every 28 days	50 mg/m^2^ cisplatin & 50 mg/m^2^ doxorubicin on day 1 repeated every 28 days	3 CR	1 PR	2 SD 1 PD	—	—
Hoskins et al. (1995)	8	Hysterectomy (3)	40 mg/m^2^ for 5 days at 14‐day intervals (5 cycles)	25 mg/m^2^ for 5 days at 14‐day intervals (5 cycles) Radiotherapy concurrently from 2nd cycle	5 CR	2 PR	1 PD	28% FFS (at 3 years)	28% (at 3 years)
Hoskins et al. (2003)	31	—	SMCC (*n* = 17)—40 mg/m^2^ for 5 days at 14‐day intervals (5 cycles) SMCC2 (*n* = 14)—75 mg/m^2^/day etoposide—100 mg oral etoposide	SMCC: 25 mg/m^2^ for 5 days at 14‐day intervals (5 cycles) Radiotherapy concurrently from 2nd cycle SMCC2: Paclitaxel (175 mg/m^2^) Cisplatin (60 mg/m^2^) Locoregional irradiation Carboplatin (area under the curve, 5 or 6)	SMCC‐12 CR (70%) SMCC2‐10 CR (70%)	SMCC‐2 PR (12%) SMCC2‐2 PR (14%)	SMCC—0 SD (0%) SMCC2–0 SD (0%) SMCC—0 PD (0%) SMCC2–1 PD (7%)	57% FFS (at 3 years same for both SMCC and SMCC2)	60% (at 3 years same for both)
Tokunaga et al. (2013)	9	No previous chemotherapy or irradiation	Etoposide (100 mg/m^2^) on day 22–24	Stage IB‐IIA patients underwent radical hysterectomy (type III or type II) and pelvic lymphadenectomy External beam pelvic radiotherapy (EBRT) was initiated within 6 weeks of surgery Vincristine (1 mg/m^2^), adriamycin (40 mg/m^2^) and cyclophosphamide (1000 mg/m^2^) on day 1, and cisplatin (100 mg/m^2^) on day 22	1 CR	2 PR	1 PD	56% PFS	52% OS
Bae et al. (2008)	99	No prior treatment	Etoposide (100 mg/m^2^) on day 1	Cisplatin (60 mg/m^2^) days 1 and 2 Type III radical hysterectomy with lymph node dissection	1 CR (1%)	68 PR (68.7%)	28 SD (28.3%) 2 PD (2%)	3‐year PFS: 77.2% 5‐year PFS: 60.5%	3‐year OS: 91.3% 5‐year PFS: 88.1%
Iwasaka et al. (1998)	16	No prior therapy	100 mg/m^2^ etoposide on days 1, 3 and 5 every 4 weeks	50 mg/m^2^ cisplatin and 10 mg/m^2^ mitomycin C on day 1 for every 4 weeks	3 CR	5 PR	8 (no change)	—	—
Al‐Saleh et al. (1997)	38	No prior chemotherapy except for cisplatin if it had been used as a radiation sensitizer	60 mg/m^2^/day etoposide intravenously, followed by 50 mg oral etoposide for 7 days, and repeated at 28‐day intervals	30 mg/m^2^/day cisplatin intravenously	7 CR	8PR	7 SD 16 PD	—	Responders: 1 year OS (65%) 2‐year OS (37%) Non‐responders: 1 year OS (6%) 2‐year OS (0%)

## 4 Adverse Effects

**FIGURE 5:**
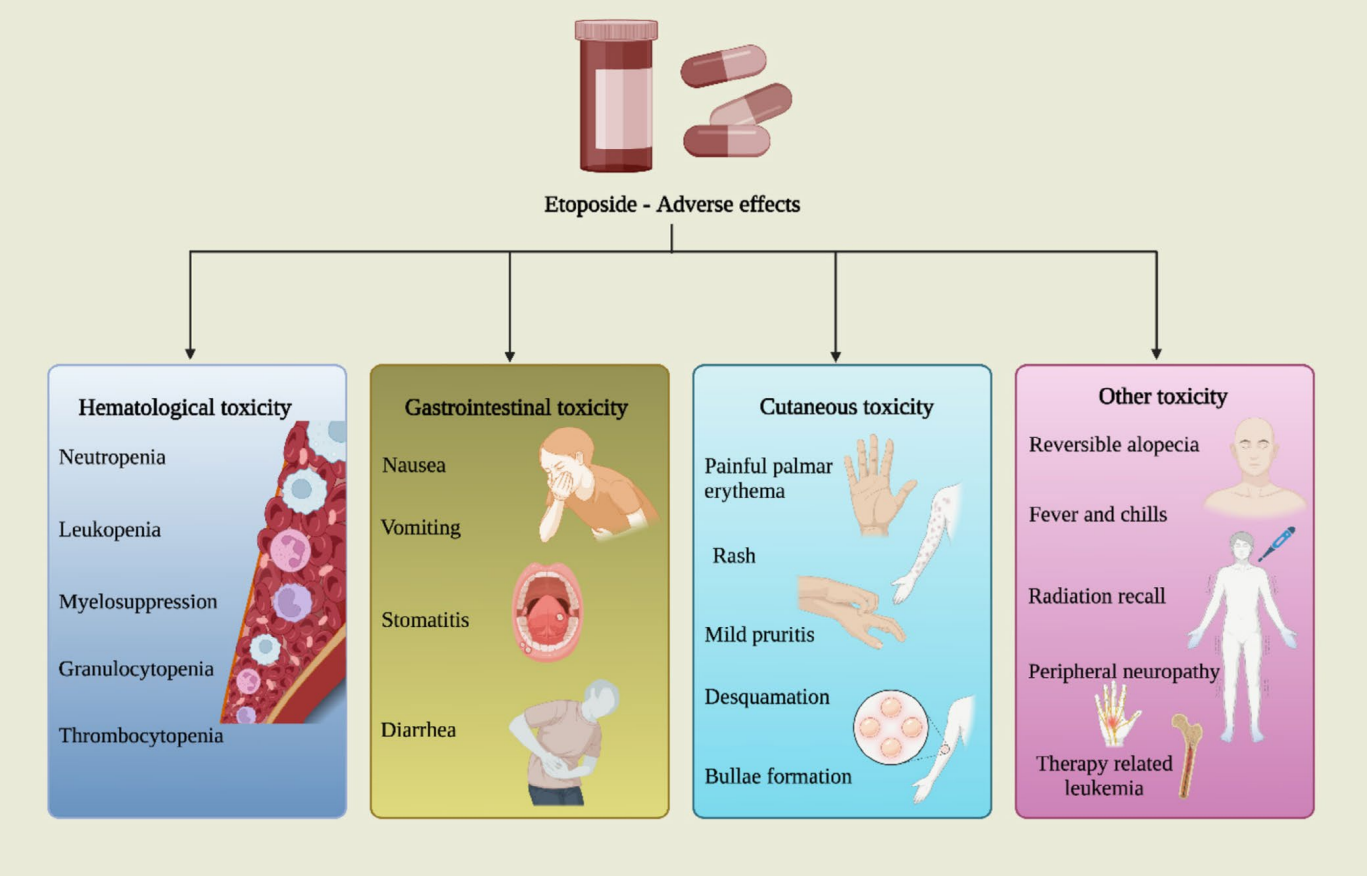
Adverse effects related to etoposide treatment.

Several studies have highlighted the adverse effects associated with oral etoposide treatment. Morris et al. reported that oral etoposide led to grade 3 or 4 granulocytopenia in 11% of patients, increasing their risk of infections. Additionally, gastrointestinal symptoms such as nausea, vomiting, diarrhea, and stomatitis were observed in 11% of cases, along with neutropenic fever (6.5%), thrombocytopenia (10%), and universal alopecia [34]. Similarly, Rose et al. [37] documented significant hematologic toxicities with second‐line oral etoposide treatment, where 33.3% of patients experienced grade 4 neutropenia, 15% had grade 4 thrombocytopenia, and 41.7% developed grade 3 or 4 anemia. Nonhematologic toxicities such as nausea and vomiting were reported in 16.7% of cases. In another study by Rose et al. [38] extended oral etoposide therapy in cervical nonsquamous cell carcinoma showed a slightly lower incidence of grade 4 neutropenia (29.8%) and thrombocytopenia (8.5%). However, severe nonhematologic toxicities, including gastrointestinal complications, grade 3 weakness, fever, and genitourinary symptoms, were reported. Furthermore, a study on continuous low‐dose etoposide in persistent and recurrent cervical cancer revealed notable toxicity profiles. Grade 2 neutropenia occurred in 33.3% of patients, while 6.6% experienced grade 3 neutropenia. Additionally, 13.3% had thrombocytopenia, and 53.3% developed anemia, making etoposide less tolerable compared to cyclophosphamide [39]. Several studies have linked etoposide to therapy‐related leukemia (t‐AML). Le Deley et al. reported that high‐dose, continuous etoposide treatment increases t‐AML risk in solid tumors. Winnick et al. found that within 23– 68 months post‐treatment, 5.9% (10/205) of children developed secondary AML. Sugita et al. observed a high incidence of secondary leukemia in children with non‐Hodgkin's lymphoma, recommending twice‐weekly dosing to reduce risk. Kollmannsberger et al. reported a 1.3% incidence of s‐AML in patients receiving etoposide doses > 2 g/m^2^, while Ratain et al. linked high‐dose etoposide (median: 6795 mg/m^2^) to leukemia in non‐small cell lung cancer [71]. A study by Murphy et al. [72] reported that a generalized, pruritic, erythematous maculopapular rash occurred in 24.8% (36/145) of patients receiving high‐dose etoposide. The most severe cases were observed in six patients, all of whom received the highest dose (4200 mg/m^2^). These cases were characterized by intense palmar erythema, initially appearing over the thenar eminence, finger sides, or knuckles, progressing to swelling, deep redness, and severe pain, followed by bullae formation and desquamation. These findings collectively indicate that oral etoposide is associated with significant hematological, gastrointestinal, cutaneous and other adverse effects (Figure 5), necessitating careful patient monitoring.

## 5 Factors Affecting Sensitivity to Etoposide

p53 is targeted for degradation by HPV oncoproteins, leading to cell cycle deregulation. The E6 and E7 oncogene products, which alter infected cells, are responsible for the carcinogenic potential of HPV types 16 and 18 and, to a lesser extent, 33, 45, 52, and 58. The p53 protein is targeted by the E6 oncoprotein, which also causes its ubiquitin‐mediated degradation and alters cell cycle regulation [73, 74]. To reestablish the apoptotic activity of these cells, Reddy et al. studied how to downregulate HPV transcription and stabilize p53. HPV‐18‐positive HeLa cells became more susceptible to etoposide‐induced apoptosis and cell cycle arrest as p53 and its target gene, Bax, get accumulated. Vitamin C was used to stabilize P53 and enhance cervical cancer cell drug sensitivity. In the absence of p53, ionizing radiation and chemicals that damage DNA do not cause G1 arrest or apoptosis. Following 36 h of priming with 1 μM vitamin C, the percentage of apoptotic cells increased from 43.1% to 66% with 2 μM etoposide [74].

Using the HPV16‐positive cervical cancer cell line, SiHa, Koivusalo and Hietanen investigated the function of p53 in mediating the cytotoxic effects of chemotherapy agents. They discovered that the drug used and the p53 status impact chemosensitivity. In cervical cancer cells, etoposide increased p53 reporter activity 10‐fold, suggesting p53 activation. Etoposide reduced E6 mRNA levels by 70%, suggesting that a reduction in E6 mRNA is required but is insufficient for activating p53. p53 inhibition sensitized SiHa cells to etoposide. In the absence of functioning p53, etoposide increased caspase 3/7 activation, and its cytotoxicity increased by approximately 30%. Since the cytotoxicity of etoposide is enhanced in functionally p53‐null cells, it is possible that these drugs are much more potent against advanced p53‐mutated cancers than against early‐stage tumors that express wild‐type p53 [75].

The normal cervical epithelium has very low levels of CD40, but lesions infected with human papillomavirus and advanced squamous cell cervical cancer have high levels of this protein. Hill et al. [76] used the cervical cancer cell lines CaSki (an HPV16‐transformed line expressing CD40) and HeLa (which do not express CD40 but are HPV18 positive and were transfected with a CD40 expression vector). Soluble CD40L stimulates CD40‐positive cervical cancer cell lines and affects their susceptibility to CTL‐mediated death. Clinically relevant amounts of etoposide were used for 16 h to treat the HeLa‐CD40 cells. After that, the cells were cocultured with 1 μg/mL CD40L for 48 h. When combined, etoposide and CD40L significantly increased apoptosis to approximately 40% in HeLa‐CD40 cells, but CD40L alone did not cause cell death, and etoposide alone had little impact. Combination therapy, which combines anticancer medications such as etoposide with adenovirus or soluble CD40L, may increase anticancer activity and thus enhance therapeutic efficacy without increasing the harmful effects that are often linked with high dosages of chemotherapy.

Tanaka et al. [77] used the human cervical squamous cell carcinoma cell line ME180 to study the best protocol for the administration of etoposide during chemotherapy and chemoradiotherapy. The etoposide sensitivity of cells was reduced in a dose‐dependent manner by concurrently administering therapeutic doses of radiation. In contrast to nonirradiated parent cells, three of the four post‐irradiation surviving subclones showed greater sensitivity. Six monoclonal etoposide‐resistant cells were established, five of which were radioresistant. In addition, these subclones showed resistance to other anticancer medications, such as pirarubicin, docetaxel, carboplatin, paclitaxel, nedaplatin, and cisplatin. However, in comparison to the parent cells, these cells showed increased sensitivity to SN38,5‐fluorouracil, and mitomycin C. These cells had significantly greater CD40 expression on the cell surface, and the expression of some integrin receptor subunits, such as CD29, CD49a, and CD49f, was reduced compared to that in the parent cell. Based on these results, the authors suggested that for advanced cervical squamous cancer patients, etoposide should be administered after the completion of radiotherapy instead of concurrent chemoradiotherapy, and mitomycin C, 5‐fluorouracil, and irinotecan are possible combination drugs that can be used with etoposide to kill resistant cells.

Isothiocyanates sensitize HeLa cells to etoposide‐induced cytotoxicity. The EC50 of etoposide in the absence of isothiocyanates was 95 μM, which was reduced to 25 μM in the presence of phenylethisothiocyanate (PEITC) and 32 μM in the presence of sulforaphane (SFN) (5 μM). The same extent of killing could be achieved at lower concentrations of the drug. SFN and PEITC are isothiocyanates. Simultaneous etoposide treatment with PEITC increased the treatment efficacy by 3.8‐fold, and SFN increased the treatment efficacy by 2.9‐fold. Pretreatment of HeLa cells with isothiocyanates before treatment with etoposide also increased the efficacy of etoposide. The apoptotic index (the ratio of apoptotic to nonapoptotic cells) also increased in both cases. Combined treatment with etoposide and isothiocyanates increases caspase 3 and 8 in HeLa cells. Pretreatment was more effective at inducing apoptosis in HeLa cells via the modification of telomerase and PKCs. In synergistic cancer therapy, this isothiocyanate may prove to be valuable for minimizing drug dosage [78].

In the cervical cancer cell line HeLa, Saito et al. [79] demonstrated that RNF4 (RING finger protein 4) is localized to mitotic chromosomes in a SUMO‐2/3‐dependent manner during etoposide treatment. In mitotic cells, RNF4 participates in the damage response triggered by etoposide by acting as a SUMO‐targeted ubiquitin ligase (STUbL). RNF4 removal increased the susceptibility of mitotic HeLa cells to etoposide treatment, and the results suggested that RNF4 is a component of the signaling cascade that prevents mitotic cells from dying when exposed to etoposide, which causes resistance to etoposide.

Compared with those in normal cervical tissues, the protein and mRNA expression levels of IER3 and TAp73b were either undetectable or extremely low in cervical cancer tissues. Jin et al. [80] proposed that cervical cancer cells become more sensitive to etoposide due to c‐Abl‐mediated upregulation of TAp73b, which eventually activates IER3.

Yuan et al. [81] showed that the cervical cancer cell lines HeLa and SiHa overexpress trefoil factor 3 (TFF3). Furthermore, overexpression of TFF3 did not affect P‐glycoprotein (P‐gp) expression but increased P‐gp functional activity, which reduced sensitivity to etoposide. TFF3 knockdown enhanced etoposide sensitivity. Therefore, TFF3 could be a therapeutic target.

Each drug has distinct pharmacokinetics and biodistributions, and preserving the precise drug ratios systemically that were previously chosen in vitro is very difficult. Thus, the drug combination can be delivered using nanocarriers to maintain an effective drug ratio. These nanocarriers have shown promising characteristics for drug delivery, such as extended duration in the bloodstream, decreased nontargeted absorption, ability to target specific cells, controlled drug release, and capacity to encapsulate multiple drugs for combination therapy [82]. Disulfide cross‐linked poly (ethylene glycol) monomethacrylate (POEOMA) nanogels were used to encapsulate vorinostat (VOR) and etoposide (ETOP). The natural reducing agent glutathione (GSH) inside the cells breaks down the nanogels. The two medications were encapsulated using a physical encapsulation method and showed a sustained release profile. Compared with that of the cells treated with the free drug, the viability of the HeLa cells treated with the individual drug encapsulated POEOMA nanogels significantly decreased. HeLa cell viability was significantly lower when the VOR‐POEOMA and ETOP‐POEOMA nanogel combinations were administered simultaneously than when free vorinostat and etoposide were administered in combination. This was because of increased caspase 3/7‐mediated apoptosis. Compared with the combination of free drugs, the coadministration of chemical agents encapsulated in nanoparticles produces synergistic effects and increases therapeutic efficacy [42].

Ruíz et al. reported that cancer stem cells (CSCs), which are derived from cervical cancer cell lines, have reduced sensitivity to etoposide (VP16) and overexpress RAD51. Resveratrol or siRNA targeting RAD51 mRNA were used to suppress RAD51 expression in CSC‐enriched cells. This resulted in reduced cell viability and the induction of apoptosis upon treatment with VP16. Resveratrol‐induced inhibition increased the susceptibility of CSCs to VP16 treatment [83].

Das et al. evaluated HAUSP and Cdc25A mRNA expression levels in various cell lines via bioinformatics analysis. The findings demonstrated that while Cdc25A is only weakly to moderately expressed in noncancerous cells, it is highly expressed in cervical cancer cells. In both malignant and noncancerous cell lines, HAUSP expression is high. HAUSP and Cdc25A are important regulators of cervical cancer development. Furthermore, the level of expression of the Cdc25A protein reduced in HeLa cells where HAUSP decreased, affecting the cells' vulnerability to medications that harm DNA. For 6 h, HeLa cells were exposed to 10 μM etoposide. HAUSP‐transfected HeLa cells were more resistant to etoposide than sham control cells, although HAUSP‐knockout cells showed greater sensitivity. From a clinical perspective, these results suggest that the Cdc25A‐HAUSP network is a viable target for lowering cancer development and treatment resistance in concert. HAUSP could be a crucial target for overcoming the defense mechanisms used by cervical cancer cells [84].

## 6 Discussion

Phase‐specific chemotherapeutic drug etoposide targets cells in the late S and early G2 stages of the cell cycle [85]. The timing of etoposide's administration has a direct bearing on its effectiveness [86]. The reasons for this include the levels of topoisomerase II, which peak in the late G2/M phase and rise prior to DNA replication. Consequently, extended etoposide therapy may be beneficial as it increases exposure to critical cell cycle phases [87]. For the treatment of cancer, oral etoposide is taken into consideration due to its extended administration, affordability, and convenience of usage. The timing and dosage dependence of etoposide's action have been confirmed by preclinical research [3]. Consequently, polymeric implants may eventually replace chemotherapy regimens requiring several doses. According to studies conducted by Solano and others, etoposide‐containing poly(ε‐caprolactone) implants have demonstrated regulated and prolonged release, cytotoxicity in vitro, and good short‐term tolerance in vivo. By delivering etoposide locally, these implants can reduce systemic toxicity and increase medication exposure to tumor cells [88].

The pharmacokinetics of the medications, which produce nonuniform distribution following systemic injection, are a key influence in the differences in the results of drug combinations in vitro and in clinical settings. This emphasizes how crucial regulated and accurate medication administration is to successful combo treatments. Drug‐encapsulating nanocarriers that enable regulated exposure sequences, decreased drug resistance, and improved therapeutic efficacy have demonstrated promise [41]. With the ability to target several locations at once, dual‐target ligands are also becoming more popular to get around the drawbacks of combination therapy, especially for multimodal illnesses like cancer [46, 89, 90, 91].

Topoisomerase inhibitor drug resistance frequently entails overexpression of alternative topoisomerases and downregulation of the target enzyme. This finding implies that inhibiting topoisomerases I and II at the same time may help reduce resistance [43]. Strong cytotoxic action is shown in vivo by the dual inhibitor tafluposide, which is produced from etoposide [46]. The combination of topoisomerase inhibitors and HDAC inhibitors shows promise since they both sensitize cancer cells and increase the effectiveness of other anticancer medications [92].

Etoposide has been investigated both alone and in conjunction with other medications for the treatment of cervical cancer. Although extended oral etoposide treatment is not very effective for advanced or recurrent cervical malignancies, persistent or recurrent cases after chemotherapy and radiation can be treated with a continuous low‐dose regimen (50 mg/day for 14 days in a 21‐day cycle). It is not as promising as second‐line therapy, though, and as a single agent is less successful than other palliative care regimens (at a dosage of 50 mg/m^2^/day for 21 days every 4 weeks). However, it provides moderate advantages to individuals with non‐squamous cell cervical cancer who have not had chemotherapy before. More study is necessary to determine whether etoposide, doxorubicin, and cisplatin may be used as an adjuvant to radiation or surgery in early stages of small‐cell cervical cancer. Since etoposide has a low toxicity profile, it can be used in palliative situations and is similar to other cisplatin‐based regimens for advanced or recurring cervical cancer. It has demonstrated promising outcomes as a neoadjuvant therapy with minimal toxicities and improved survival rates. A better prognosis was shown in patients who underwent adjuvant chemotherapy along with at least five cycles of EP. Targeting proteins linked to etoposide resistance in cervical cancer cell lines may also improve the cancer cells' susceptibility to etoposide‐induced cytotoxicity. Various factors influence the etoposide sensitivity, it is crucial to recognize that most observations stem from preclinical cell‐based studies. The translational relevance of these findings to clinical settings remains uncertain, as in vitro models do not always accurately predict clinical outcomes. Therefore, further clinical research is needed to validate these results and optimize etoposide‐based treatment strategies in cervical cancer therapy.

Continued investigation into enhanced drug delivery techniques and combination strategies is necessary to maximize the therapeutic potential of etoposide in the treatment of cervical cancer.

## Author Contributions

**Nikitha Kotian:** writing: original draft; review and editing. **Yashaswini Reddy:** writing: review and editing; **Padmini Pai:** visualization; writing: review and editing. **Babitha Kampa Sundara:** conceptualization; visualization; supervision; writing: review and editing.

## Funding

The work was supported by Intramural funding (Grant ID: MAHE/CDS/PHD/IMF/2023), MAHE, Manipal and MRB Seed grant (Grant ID: DOR/MRB/2023/SG‐03), MAHE, Manipal for funding.

## Ethics Statement

The authors have nothing to report.

## Conflicts of Interest

The authors declare no conflicts of interest.

## Data Availability

The authors have nothing to report.
